# Increased autophagy/mitophagy levels in primary tumours of patients with pancreatic neuroendocrine neoplasms

**DOI:** 10.1007/s12020-020-02228-1

**Published:** 2020-02-29

**Authors:** Kosmas Daskalakis, Krystallenia I. Alexandraki, Ismini Kloukina, Evanthia Kassi, Evangelos Felekouras, Evangelia Xingi, Stamatis N. Pagakis, Apostolos V. Tsolakis, Evangelos Andreakos, Gregory Kaltsas, Konstantinos Kambas

**Affiliations:** 1 1st Department of Propaupedic Internal Medicine, Endocrine Oncology Unit, Laiko Hospital, National and Kapodistrian University of Athens, Athens, Greece; 2 Department of Surgery, Faculty of Medicine and Health, Örebro University, Örebro, Sweden; 3 Center of Basic Research, Biomedical Research Foundation of the Academy of Athens, Athens, Greece; 4 Department of Biological Chemistry, Medical School, National and Kapodistrian University of Athens, Athens, Greece; 5 First Department of Surgery, Laikon General Hospital, University of Athens Medical School, Athens, Greece; 6 Microscopy Unit, Hellenic Pasteur Institute, Vas. Sofias 127, Athens, 11521 Greece; 7 Biological Imaging Unit, Biomedical Research Foundation of the Academy of Athens, Athens, 11527 Greece; 8 Department of Oncology and Pathology, Karolinska Institute, Solna R8:04, Stockholm, 17177 Sweden; 9 Laboratory of Immunobiology, Center for Clinical, Experimental Surgery and Translational Research, Biomedical Research Foundation of the Academy of Athens, 11527 Athens, Greece; 10 Laboratory of Molecular Genetics, Department of Immunology, Hellenic Pasteur Institute, Athens, Greece

**Keywords:** Pancreatic neuroendocrine neoplasms, Autophagy, Mitophagy

## Abstract

**Background/aims:**

We assessed the levels of autophagy and mitophagy, that are linked to cancer development and drug resistance, in well differentiated pancreatic neuroendocrine neoplasms (PanNENs) and correlated them with clinico-pathological parameters.

**Methods:**

Fluorescent immunostaining for the autophagy markers LC3Β and p62/or LAMP1 was performed on 22 PanNENs and 11 controls of normal pancreatic tissues and validated through Western blotting. Autophagy quantitative scoring was generated for LC3B-positive puncta and analysed in relation to clinico-pathological parameters. TOMM20/LC3B qualitative assessment of mitophagy levels was undertaken by fluorescent immunostaining. The presence of autophagy/mitophagy was validated by transmission electron microscopy.

**Results:**

Autophagy levels (LC3B-positive puncta/cell) were discriminative for normal vs. NEN pancreatic tissue (*p* = 0.007). A significant association was observed between autophagy levels and tumour grade (Ki67 < 3% vs. Ki67 ≥ 3%; *p* = 0.021), but not functionality (*p* = 0.266) size (cut-off of 20 mm; *p* = 0.808), local invasion (*p* = 0.481), lymph node- (*p* = 0.849) and distant metastases (*p* = 0.699). Qualitative assessment of TOMM20/LC3B demonstrated strong mitophagy levels in PanNENs by fluorescent immunostaining as compared with normal tissue. Transmission electron microscopy revealed enhanced autophagy and mitophagy in PanNEN tissue. Response to molecular targeted therapies in metastatic cases (*n* = 4) did not reveal any patterns of association to autophagy levels.

**Conclusions:**

Increased autophagy levels are present in primary tumours of patients with PanNENs and are partially attributed to upregulated mitophagy. Grade was the only clinico-pathological parameter associated with autophagy scores.

## Introduction

Pancreatic neuroendocrine neoplasms (PanNENs) are relatively rare and comprise a heterogeneous group of neoplasms with diverse biological behaviour and a wide range of response to existing therapies [1]. The current tumour classifications and NEN grading system do not allow for the timely identification of patients with tumours refractory to available multi-modal treatment. Although molecular targeted therapies (MTTs), such as mammalian target of rapamycin (mTOR) inhibitors and tyrosine kinase inhibitors, have been shown to improve outcomes in patients with metastatic disease, resistance to molecular targeted therapies often occurs [2, 3]. Identification of predictive markers of response, as well as understanding of the mechanisms involved in NEN oncogenesis, metastasis and treatment resistance could potentially facilitate the development of novel therapeutic approaches and guide the selection of existing targeted therapies.

Autophagy is a key homoeostatic machinery of cellular self-degradation, highly evolutionary conserved, that has recently been involved in the pathophysiology of oncogenesis and anti-cancer treatment resistance [4]. Degraded cellular components are engulfed into autophagosomes and fusion of the autophagosomes with lysosomes forms autolysosomes [5]. In normal conditions, autophagy is generally maintained at a constant, basal activation rate, whereas in cases of nutrient deprivation, as in hypoxia and DNA damage, the mechanism of autophagy is further activated, acting as an anti-apoptotic pathway [6–8]. Recycling of cellular components from autolysosomes provides an important source of amino acids, nucleotides and lipids to neoplastic cells. Interestingly, across different tumour types, autophagy may exhibit promoting or inhibitory effects to carcinogenesis by indorsing resistance to anticancer therapies or inducing tumour cell cycle arrest, respectively [9–11].

In some cancer types autophagy activity has been linked with tumour aggressiveness and poor patient outcomes [12, 13]. However, the exact interactions of certain molecular pathways in NENs, such as the mTOR and the angiogenesis pathways, with autophagy and mitophagy have not been sufficiently elucidated. Furthermore, no potential relations of autophagy markers with NEN clinico-pathological parameters have been evaluated. Inactivation of the mTOR pathway is caused by various factors that are involved in the energy cycle of the neuroendocrine cell. The mTOR negatively regulates autophagy by phosphorylating and inactivating Ulk1, a serine/threonine kinase that acts at the onset of autophagy. PI3K/Akt/mTOR inhibitors initiate autophagy by promoting survival, that may interfere with their anticancer activity. Therefore, autophagy inhibition is used as a strategy to enhance the efficacy of PI3K/Akt /mTOR inhibitors in different cancers [14].

With regards to angiogenesis, the multi-kinase inhibitor sunitinib has emerged as a promising agent in PanNENs targeting neo-vascularisation via inhibiting VEGFR, PDGFR, and c-KIT. Anti-angiogenic therapy might induce autophagy in both tumour cells and microenvironment [15]. Interestingly, while the activation of autophagy in cancer cells retards their proliferation, stromal cancer-associated fibroblasts auto-digesting themselves into basic degraded nutrients may promote systemic dissemination [16]. Hence, diverge effects of anti-angiogenics on primary tumour and its microenvironment may be present in different stages of NEN development and been altered by different therapeutic regimens [17]. To date, two ex vivo studies on PanNENs have demonstrated that co-administration of the autophagy inhibitor chloroquine to everolimus and sunitinib increases MTT efficacy [18, 19]; however studies on humans are currently missing.

Mitophagy is the selective autophagic degradation of impaired mitochondria and has also been associated to oncogenesis [20]. One of its main mediators, Parkin, a putative tumour suppressor gene, is translocated to the mitochondria secondary to loss of mitochondrial membrane potential and ubiquitinates mitochondrial proteins, recruiting p62, LC3 and autophagosomes to the mitochondria [20, 21]. Mitophagy is involved in tumour resistance to cancer treatment by removing damaged mitochondria and maintaining functional ones [22]. Currently, a mechanism of mitophagy based on PTEN-induced putative kinase 1 (PINK1) and Parkin is widely accepted. Antiangiogenic agents, such as sunitinib trigger mitochondrial damage, cytochrome C release, caspase 9 activation and apoptotic cell death both in vitro and in vivo [23]. However, it remains to be determined whether angiogenesis inhibitors modulate mitophagy and if therapeutic intervention with mitophagy could sensitise cancer cells to these agents [24].

In the present study, we investigated the levels of autophagy by means of immunofluorescence/confocal microscopy, immunoblotting and transmission electron microscopy in primary PanNENs as compared with normal pancreatic tissues, as well as the potential association of these with certain clinico-pathological parameters and clinical response to MTT. In addition, we investigated the levels of mitophagy present in PanNENs with the aims to highlight whether the presence of mitophagy may contribute to overall autophagic activity in these tumours.

## Methods

### Sample collection

**Fig. 1:**
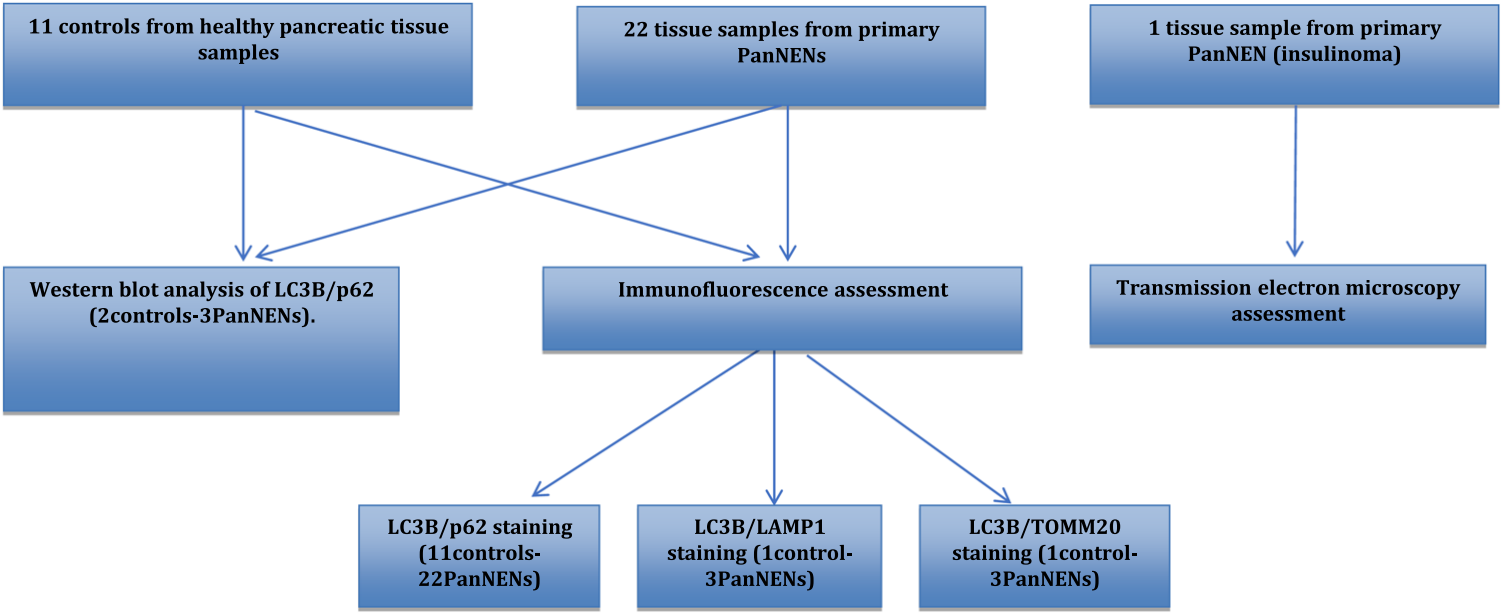
Study flow chart

Retrospectively, 22 histological samples from surgically removed primary PanNENs as well as 11 histological samples from normal pancreatic tissue (healthy resection margins of non-neuroendocrine pancreatic lesions) were obtained from the pathological archive of the EKPA-Laiko University Hospital, Athens, Greece. Fresh tissue from the central part of the tumour of three PanNENs and the healthy resection margin of two controls of normal pancreatic tissue was also obtained for validation experiments from these 33 cases (Fig. 1). The study protocol design was in accordance with the Declaration of Helsinki, and the procedures have been approved by the local ethics committee of the EKPA-Laiko University Hospital, Athens, Greece (Drn:15161). Written consent was obtained for all analysed patient tissue specimens. We used the 2017 WHO classification systems for grading gastro-enteropancreatic NENs [25]. For staging, we used the 8th edition of the American Joint Committee on Cancer [26]. Demographic and clinical characteristics including tumour functionality, histology and TNM stage are presented in Table 1.

**Table 1:** Baseline characteristics of patients with pancreatic neuroendocrine neoplasms

	Number of patients (%)
Age (years; median with range)	52 (39–82)
Gender	
Female	11 (50)
Male	11 (50)
Sporadic disease	
No	0
Yes	22
Functionality	
Non-functional tumour	15 (68)
Insulinoma	5
Gastrinoma	2
Grading	
Grade 1 (Ki67 < 3%)	11 (50)
Grade 2 (Ki67 3–20%)	9
Grade 3 (Ki67 > 20%)	2
Tumour size	
<20 mm	11 (50)
≥20 mm	11 (50)
Local invasion	
No	19 (86)
Yes	3
Lymph node metastases	
No	16 (73)
Yes	6
Distant metastases	
No	16 (73)
Yes	6
SSA	
No	15 (68)
Yes	7
MTT	
No	18 (82)
Yes	4

*MTT* molecular targeted therapy, *SSA* somatostatin analogue

Six patients had distant metastases, mainly to the liver at diagnosis; four out of these patients had also lymph node metastases at histopathology, whereas two patients in our cohort had locoregional lymph node metastases only found at baseline surgery. All original histological diagnoses were reviewed by a dedicated NEN pathologist. Primary treatment of PanNEN patients ranged from enucleation, distal pancreatectomy, pylorus-preserving pancreaticoduodenectomy to total pancreatectomy. In patients with advanced locoregional disease or distant metastases (*n* = 4), systemic treatment consisting of MTT was administered as per ENETS guidelines [27]. Both archival and fresh pancreatic tissues were obtained before treatment initiation, i.e. primary tumour samples were evaluated at disease diagnosis and before any treatment. Information on tumour treatment and follow-up, including the use of mTOR and/or anti-angiogenic treatment, were retrospectively retrieved from the patient’s records. Disease status during follow-up was defined using the RECIST 1.1 criteria [28].

### Immunofluorescence

Representative areas from the central part of the tumour were identified by a dedicated NEN pathologist. Five-micrometre consecutive tissue sections were cut from each paraffin block and prepared on pathological slides. Immunofluorescence experiments were performed in formalin-fixed, paraffin-embedded blocks that were routinely prepared from the surgical specimens of PanNENs and controls. Immunofluorescence protocol for autophagy detection was performed as previously described [29, 30]. Non-specific binding sites were blocked by incubation with 2% normal goat serum in PBS-Triton X100 0.2% for 45 min. Then the sections were incubated overnight at 4 °C with a polyclonal rabbit anti-human LC3B antibody (1:200; Sigma-Aldrich, L7543) and a monoclonal mouse anti-human p62 ([SQSTM1]1:300; MBL, ab8878). A mouse anti-TOMM20 antibody (ab56783) or a-mouse anti-LAMP1 antibody - Lysosome Marker (ab24170) in combination with the rabbit anti-LC3B were also used in the same fashion in limited samples (*n* = 4) for qualitative analysis. A goat anti-rabbit Alexa Fluor 594 antibody (Abcam, ab150080) and a mouse anti-rabbit Alexa Fluor 488 antibody (Abcam, ab150113) were used as secondary antibodies (1:400 in blocking solution). Appropriate rabbit and mouse IgGs were used as negative controls. Immunofluorescence was observed and scanned with a ×40 objective using the Leica TCS-SP8 Confocal Microscope of the Light Microscopy Unit of the Hellenic Pasteur Institute. Colocalization of Abs (LC3B, p62, LAMP-1, TOMM20) and calculation of autophagy scores, defined as the numbers of LC3 puncta/cell were performed as previously described [29, 30], using a macro developed in Fiji software [31]. All measurements were done only in the tumour area in PanNENs or in normal pancreatic tissue, not considering any positive signal in stroma-surrounding areas.

### Western blot analysis

PanNEN tissue specimens were homogenised and quantified for total protein content according to standard protocols. To study autophagy, western blotting was performed as previously described [32]. In brief, a rabbit anti-human LC3B polyclonal antibody (1/1000 dilution; Sigma-Aldrich, L7543) and a mouse anti-human p62/SQSTM1 monoclonal antibody (1/750 dilution; MBL, ab8878) were used. To verify equal loading in cell lysates (40 μg), membranes were re-probed for GAPDH (1/1000 dilution; Millipore, MAB374).

### Transmission electron microscopy

For conventional electron microscopy, fresh PanNEN tissue from an insulinoma was cut into small blocks and fixed in 2.5% glutaraldehyde made up in 0.1 M Phosphate buffer solution (PB), pH 7.4 for 24 h. After washing with 0.1 M PB, the specimens were post-fixed with 1% osmium tetroxide for 1 h. They were then dehydrated and embedded in Epon/Araldite resin mixture and allowed to polymerise at 60 °C for 24 h. Ultrathin sections were cut with a Diatome diamond knife at a thickness of 65 nm on Leica EM UC7 ultramicrotome (Leica Microsystems, Vienna, Austria), were then collected onto 300 mesh nickel grids and stained with uranyl acetate and lead citrate. Sections were examined with a Philips 420 transmission electron microscope at an acceleration voltage of 60 kV and photographed with a Megaview G2 CCD camera (Olympus SIS, Münster, Germany).

## Statistical analysis

Statistical analyses were performed using two-tailed Student’s *t* test with GraphPad Prism version 8.0.0 for Windows, GraphPad Software, San Diego, CA, USA, www.graphpad.com. Data are presented as mean ± SD. Differences were considered statistically significant at *P* < 0.05. All data were analysed using GraphPad Prism v8 software.

## Results

### Increased autophagy levels in PanNEN tissue specimens

**Fig. 2:**
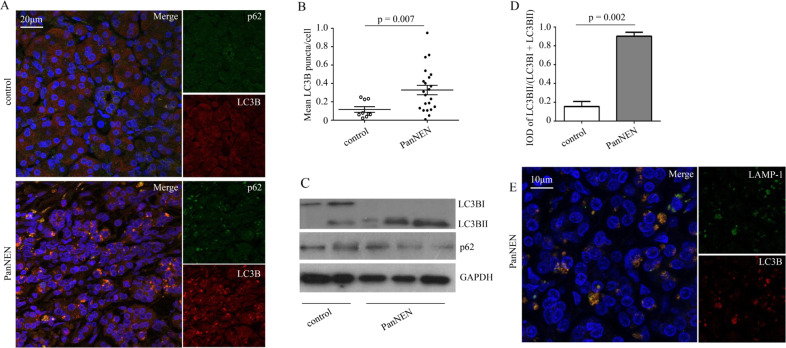
Increased autophagy in pancreatic neuroendocrine neoplasm (PanNEN) tissues. **a** Immunofluorescent staining of LC3B (red), p62 (green), and DAPI (blue) in primary PanNEN (*n* = 22) and normal pancreatic tissue (control, *n* = 11). Scale bars: 20 μm; ×40 objective. **b** Quantitative analysis of LC3B-positive puncta in immunofluorescence images. Means ± SD LC3B puncta/cell are depicted. *P* value has been calculated by the student’s *t* test. **c** Western blot analysis of LC3B and p62 in PanNENs (*n* = 3) and controls (normal pancreatic tissues; *n* = 2). GAPDH was used as loading control. **d** Integrated optical density (IOD) of LC3BII/(LC3BI + LC3BII) immunoblotting between PanNENs and controls is depicted. Results are expressed as mean ± SD. Statistical analysis was performed using student’s *t* test. **e** Immunofluorescent staining of LC3B (red), LAMP-1 (green), and DAPI (blue) in in PanNENs (*n* = 3) and control tissue (*n* = 1). Scale bar: 10 μm; ×63 objective. One representative out of five experiments

To investigate the autophagic levels in neuroendocrine neoplasms, we assessed LC3B and p62 expression in paraffin sections of PanNENs (*n* = 22) and normal pancreatic tissue specimens (*n* = 11), via immunofluorescence. PanNENs demonstrated increased LC3B puncta formations and colocalization of p62 compared to controls (Fig. 2a, b). Western blot analysis confirmed that LC3B expression was present at higher levels in PanNENs than in normal pancreatic tissue (Fig. 2c, d). Comparison of integrated optical density (IOD) of LC3BII/(LC3BI + LC3BII) between PanNENs and controls revealed significant higher IOD levels in PanNENs (*p* = 0.002; Fig. 2d). Moreover, in order to investigate autolysosome formation and the presence of complete autophagy, LC3B/LAMP-1 immunofluorescence staining was performed and demonstrated colocalization of LC3B and LAMP-1 (PanNENs [*n* = 3] vs. control [*n* = 1]; Fig. 2e), indicating the generation of complete autolysosomes in PanNENs. These findings indicate that primary PanNENs have increased autophagy levels, potentially implying higher autophagic induction and autophagic flux in tumour regions.

### Excessive autophagy in PanNENs includes mitophagy

**Fig. 3:**
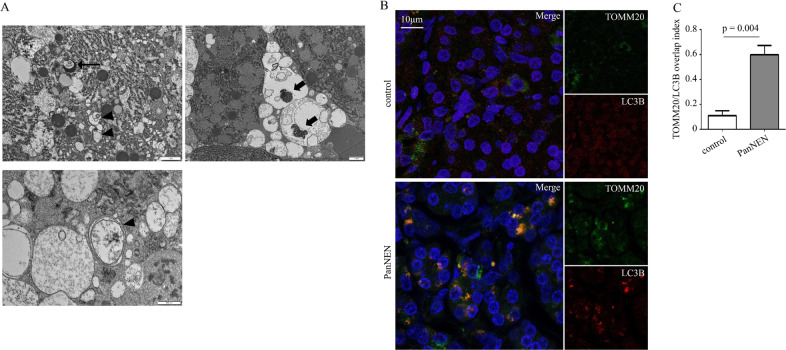
Pancreatic neuroendocrine neoplasms (PanNENs) demonstrate high levels of mitophagy. **a** Transmission electron microscopy of primary tumour from a patient with an insulinoma. Black arrowheads: autophagosomes; arrow: autolysosome; thick arrow: degraded mitochondria in autophagosomes. Scale bars depicted on each image. **b** Immunofluorescent staining of LC3B (red), TOMM20 (green), and DAPI (blue) in a PanNEN primary and control tissue. Scale bars: 10 μm, ×63 objective. One representative out of four experiments is shown. **c** Overlap/colocalization index of LC3B/TOMM20 in PanNENs (*n* = 3) and control tissue (*n* = 1). Statistical analysis was performed using student’s *t* test

To further validate these results, we performed transmission electron microscopy in fresh tumour tissue obtained from one additional patient with insulinoma. This demonstrated the presence of numerous double-membraned autophagosomes (Fig. 3a, black arrow head) undergoing lysosomal fusion (Fig. 3a, arrow), confirming active autophagy. Moreover, it was evident that autophagosomes also included degraded mitochondria (Fig. 3a, thick arrow) along with impaired mitochondrial morphology in the tumour, suggesting the presence of mitophagy. To assess this, we investigated the levels of mitophagy present in PanNENs, through immunofluorescence of LC3B/TOMM20. This indicated increased colocalization of LC3B and TOMM20 in PanNENs (*n* = 3) compared with control (*n* = 1) with TOMM20/LC3B overlap index being significantly higher in primary tumour tissue (*p* = 0.004; Fig. 3b, c). Thus, the increased autophagy present in PanNENs is partially attributed to the increased levels of mitophagy.

### Autophagy levels correlate with tumour grade in PanNENs

**Fig. 4:**
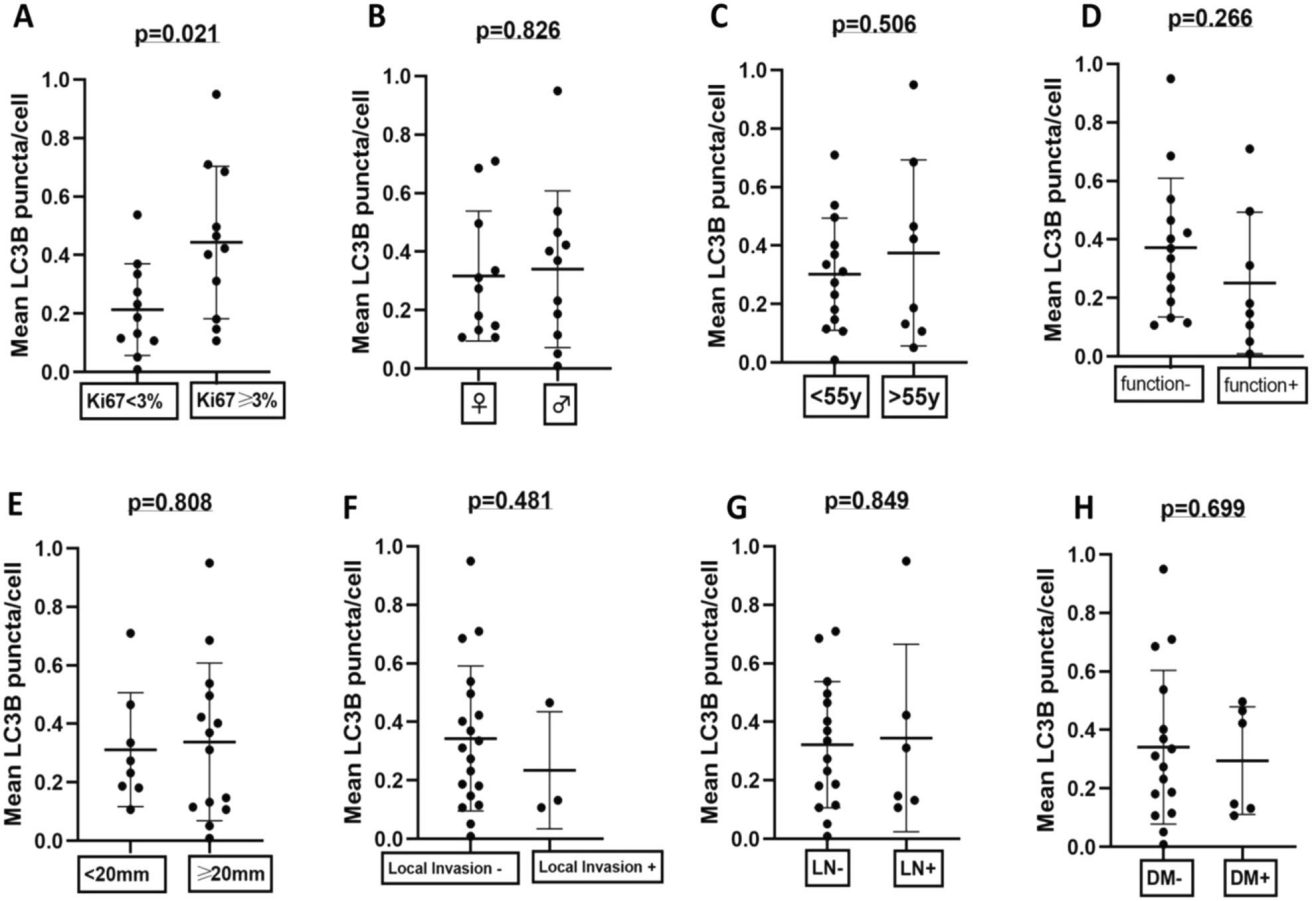
Subgroup analysis in patients with pancreatic neuroendocrine neoplasm (PanNEN) indicate correlation of autophagy levels with tumour grade. Correlation of LC3B-positive puncta scores with various clinico-pathological parameters: **a** grade (Ki67 < 3% vs. Ki67 ≥ 3%), **b** gender, **c** age (<55 years vs. ≥55years), **d** functionality, **e** tumour size (<20 mm vs. ≥20 mm), **f** local invasion, **g** nodal metastasis (lymph node negative vs. lymph node positive) and **h** distant metastasis (distant metastases negative vs. distant metastases positive)

To assess whether autophagy levels were related to certain clinico-pathological parameters and certain patient outcomes, we evaluated their correlation with gender, age, functionality, grade, tumour size, local invasion, nodal and distant metastasis and treatment response, such as progression-free survival in metastatic cases receiving everolimus or sunitinib. Interestingly, autophagy levels were elevated in patients demonstrating Ki67 ≥ 3% (*p* = 0.021, Fig. 4a), whereas no significant associations were observed between gender (*p* = 0.826), age (dichotomous division at 55 years; *p* = 0.506), functionality (*p* = 0.266), tumour size (cut-off of 20 mm; *p* = 0.808), local invasion (*p* = 0.481), lymph node- (*p* = 0.849), distant metastases (*p* = 0.699) and autophagy levels (Fig. 4). The number of metastatic cases receiving MTT (*n* = 4) precluded any safe conclusions to be made. Among the three patients with distant-stage disease that received adjuvant sunitinib, the range of autophagy scores was 49.6, 46.5 and 10.7% and only the latter exhibited disease progression at 10 months, whereas the first two patients were stable 15 and 6 months respectively after surgery and under sunitinib. One patient only received everolimus, demonstrated an autophagy score of 14.7% and had stable disease, 19 months after treatment initiation.

Hence, among the clinico-pathological parameters investigated here, tumour grade was the only factor associated with autophagic activity, whereas assessment of the clinical responses to MTTs could not reveal any patterns of association to autophagy levels.

## Discussion

Herein, we demonstrated the upregulated autophagy and mitophagy expression in PanNENs as compared with normal pancreatic tissue by the presence of specific immunofluorescence markers, been also validated at immunoblotting and transmission electron microscopy on fresh tissue. Our findings indicate that PanNENs have increased autophagic induction and autophagic flux in tumour regions and suggest that the increased autophagy levels observed are partially attributed to upregulated mitophagy. Among the clinico-pathological parameters tested, tumour grade was correlated with autophagy activity, as reflected by LC3B-positive puncta per cell scores in PanNEN primaries (*p* = 0.021). Correlations of autophagy scores with treatment responses to mTOR inhibitor everolimus and the multi-kinase inhibitor sunitinib in the few metastatic cases receiving adjuvant treatment with these agents did not reveal any patterns of altered autophagic activity with respect to its predictive ability.

The high autophagy scores in patients with higher grade neoplasms might provide additional insights into the role of autophagy in well-differentiated (WD) grade-2 and grade-3 PanNENs as opposed to grade-1 neoplasms. This finding could lead to the hypothesis that higher grade tumours might deploy the autophagy pathway to maintain their state and develop resistance to MTT [18, 33]. Notably, correlation of autophagy levels with clinicopathological parameters has not been confirmed in other cancer forms [34]. Therefore, although autophagy may be an effective target for some cancer types, it is certainly not a universally applicable approach. In addition, few patients only in our cohort received MTT postoperatively; hence, a potential association of autophagy activity with the clinical response to MTT could not be substantiated in the present study. Nevertheless, significant differences were observed when comparing autophagy scores between PanNENs and normal pancreatic tissue, thus validating the findings of ex vivo studies and potentially implying that the presence of autophagic activity may be an important player in higher grade neuroendocrine neoplasia. However, contradictory findings with biphasic modulation of autophagy by sunitinib has been reported with autophagy inhibition in response to tolerated sunitinib doses and autophagy upregulation in PanNEN cells challenged with cytotoxic sunitinib doses [35]. Therefore, identification of underlying cellular mechanisms that may upregulate autophagy in higher grade PanNENs requires further analysis in ex vivo studies on NEN cell lines as well as delineation of novel molecular prognostic markers linked with differential autophagy expression [17]. In addition, although autophagy has already been demonstrated to promote a well-differentiated state in endocrine tumours, as in thyroid cancer [36], our study confirmed that within WD tumours, higher autophagy levels were mainly found in grade 2 PanNENs. With respect to clinico-pathological correlations with autophagy scores in the aforementioned study, autophagic activity strongly correlated with clinical response to systemic treatment with radioiodine, potentially by its capacity to maintain tumour cell differentiation [36].

Several techniques have been developed to assess autophagic flux, with the aims not only to enhance sensitivity and provide a means of quantification, but also to accurately reflect the dynamic character of the autophagy multi-step pathway [37]. These include the use of the mCherry-LC3 transgenic mouse model, photo-activatable fluorescent probes and the recently described single-cell fluorescence live-cell imaging-based approach that allow for accurate autophagic flux measurements [37]. Therefore, although our findings in primary PanNENs may imply higher autophagic induction and autophagic flux in tumour regions derived from immunofluorescence co-localisation studies, we were not able to provide exact autophagic flux quantification.

MTT with everolimus or sunitinib has been approved as a part of the standard multimodal care for metastatic PanNEN patients in need of systemic treatment. The major therapeutic challenge in some of the patients is the presence of innate resistance to MTT and the selection of the ideal agent leaving patients at high risk of disease recurrence or progression. Although the mechanisms underlying MTT resistance have not been fully explained, autophagy has been demonstrated to play a pivotal role in determining cell fate across different cancer diagnoses by directing pathways of proliferation and differentiation [38, 39]. mTORC1 is a well-established inhibitor of autophagy by its direct phosphorylation and suppression of the ULK1 kinase complex [40–42]. Morover, mTORC1 inhibition using rapamycin stimulates autophagosome and autolysosome generation [43]. Therefore, NEN cells may use mTOR drug-induced inhibition to induce autophagy, enabling tumour survival and development of escape mechanisms and mTOR resistance [40, 44]. Autophagy inhibition by chloroquine alone or in combination with mTOR inhibitors was found to facilitate the antitumour effect of mTOR inhibitors in PanNEN cell lines [18, 33]. Unexpectedly, a decrease in autophagy activity was observed in a recent study in resistant NEN cell lines [45]. This decrease might be a mechanism of adaption to the permanent induction of autophagy in the presence of the mTORC1 inhibitor everolimus in line with our findings on increased autophagy levels in PanNENs prior to treatment initiation; indicating a sensitive balance of the dual effects of autophagy as a protective mechanism for the neuroendocrine cancer cell on the one hand and as a mediator of cell death on the other [46].

Mitophagy is in fact a type of selective autophagy that promotes mitochondrial turnover with the aims to maintain cellular homoeostasis. Recent reports imply that mitophagy may contribute to anticancer treatment efficacy or resistance development. However, its role across different cancer diagnoses and anticancer treatments is ambiguous. Ubiquitination of mitochondrial proteins, including TOMM20, facilitates the recognition of impaired mitochondria [47]. Blocking mitophagy has been shown to sensitise drug-resistant cancer cells to novel targeted agents [48]. Therefore, the present study of mitophagy expression in PanNENs may have important implications for the development of therapeutic agents for multidrug-resistant tumours. Our qualitative assessment of LC3B/TOMM20 and transmission electron microscopy findings in PanNENs, revealed strong mitophagy activity in these tumours, warranting further studies on mitophagy inhibition of pancreatic neuroendocrine neoplastic cells.

Our study has some limitations. The scarcity of patients with metastatic PanNENs subjected to upfront resection of the primary tumour prior to initiation of systemic treatment deems any effort to undertake a prospective study on patient outcomes and MTT responses in relation to autophagy scores or even acquire an adequate sample size a difficult task. Indeed, due to the small sample size of this study, subgroup analysis findings should be interpreted with caution. In particular, with respect to treatment response, prospective paired tissue sampling was not performed, as all specimens were surgically removed primaries and rebiopsied locoregional or distant recurrence sites after systemic treatment was not available during the study period. A further methodological limitation could be the lack of autophagic flux measurement, as both a block of autophagy and/or an increased flux might lead to increased autophagy scores necessitating further mechanistic validation. As our experiments were not performed on beta cell lines but human samples, both exocrine and endocrine normal pancreas was present in our controls. As a result, selective immunostaining of pancreatic islet cells in normal pancreatic tissue for quantitative assessment of autophagy scores and also assessment by immunoblotting was not feasible. Notably, autophagic induction was homogenous in normal pancreatic tissues across the whole biopsy, implying no differences in endocrine and exocrine tissues with respect to autophagy levels. Finally, our study provides a comprehensive analysis of autophagy levels in primary tumours of patients with PanNENs, following a validated methodology protocol on autophagy assessment and in relation to certain clinico-pathological parameters of known prognostic significance.

Future efforts should be directed in understanding the mechanisms of autophagy and mitophagy in PanNENs to assess whether potent autophagy inhibitors could sensitise refractory metastatic tumours to systemic (SSAs/MTTs) and liver-targeted agents (PRRT/SIRTEX). Such studies could aim to elucidate the differences in inhibiting the early vs. the late phases of autophagy, and determine normal tissue toxicity with potent autophagy inhibitors as well as the optimal duration of inhibition for tumour sensitisation to different treatments. In addition, prospective paired sampling from liver metastases in patients with stage IV disease who received MTTs would be a potential extension of this study by staining with pAkt and p70S6K antibodies to elucidate the effects of mTORi on PanNEN autophagy. These future directions in the field of autophagy will guide the effective translation of autophagy inhibition strategies to patients with PanNENs and yield valuable information regarding the role of autophagy with respect to treatment response.

In conclusion, the present human study provides evidence for active autophagy in primary tumours of patients with PanNENs, and its correlation with tumour grade, as well as the presence of high mitophagy levels in these tumours. Future analyses are warranted to assess whether the observed treatment responses to currently approved mTOR and multi-kinase inhibitors are correlated with the degree of autophagic activity in PanNENs and to delineate the design of studies with autophagy and mitophagy inhibitors in PanNEN patients. Analyses across a broader spectrum of NEN diagnoses may also be necessary to consolidate the autophagy/mitophagy effects in NEN oncogenesis and treatment resistance and implement blockade or upregulation of this pathway in NEN therapeutics.
